# Reporting and Interpreting Working Memory Performance in *n*-back Tasks

**DOI:** 10.3389/fpsyg.2017.00352

**Published:** 2017-03-07

**Authors:** Adrian Meule

**Affiliations:** ^1^Department of Psychology, University of SalzburgSalzburg, Austria; ^2^Center for Cognitive Neuroscience, University of SalzburgSalzburg, Austria

**Keywords:** working memory, *n*-back task, emotional stimuli, reaction times, accuracy

Working memory is an executive function, which involves holding information in mind and mentally working with it (Diamond, 2013). A widely used measure for the assessment of working memory function is the *n*-back task (Owen et al., 2005). Here, participants are typically instructed to monitor a series of stimuli and to respond whenever a stimulus is presented that is the same as the one presented *n* trials previously. Common versions are 2-back and 3-back tasks, in which participants have to respond to stimuli that have been presented two or three trials earlier. Zero-back and 1-back versions are also often used as control conditions.

In most studies, participants are required to respond with a button press to the relevant stimuli (= targets) and to withhold responses to distractor stimuli (= non-targets). Yet, there are also studies, in which participants are required to indicate for each trial whether the stimulus represents a target or a non-target (e.g., by pressing two different buttons; Jonides et al., 1997; Carlson et al., 1998; Perlstein et al., 2003; Harvey et al., 2005; Miller et al., 2009). Stimuli in classical *n*-back tasks are numbers or words, but pictorial versions, which display, for example, emotional scenes (Marx et al., 2011; Hur et al., in press), faces (Cromheeke and Mueller, 2016), or food (Meule et al., 2012; Meule, 2016) have also been used in recent years.

As dependent variables, most studies report response latencies (= reaction times) and accuracy (in %) or the number of errors. With increasing task difficulty (i.e., with increasing *n*s), reaction times usually increase and accuracy decreases (e.g., Jonides et al., 1997; Carlson et al., 1998; Perlstein et al., 2003; Harvey et al., 2005; Miller et al., 2009; Schmidt et al., 2009). Similarly, reaction times and accuracy are usually negatively correlated (e.g., Carter et al., 1998). In other words, higher reaction times are associated with a higher number of errors. Although this relationship exists, it appears that reaction times and accuracy have dissociable correlates. For example, Jaeggi et al. (2010) examined various *n*-back tasks and found several dissociations between reaction times and accuracy. For instance, higher accuracy (but not reaction times) in visuospatial, auditory, and dual 3-back tasks was correlated with higher fluid intelligence as measured with the Raven test. In visuospatial *n*-back tasks, reaction times (but not accuracy) were associated with reading span and digit span forward performance.

In addition to these findings, a recent study by Hur et al. (in press) further highlights the role of reaction times vs. accuracy. In that study, pictures of emotional scenes were used in a 0-back task (labeled as perception task) and a 2-back task (labeled as working memory task). The authors argued that there was a ceiling effect in accuracy and more meaningful variation in reaction times in the perception task and, thus, they focused on interpreting reaction time results. In the working memory task, however, there was more variability in accuracy and less variability in reaction times and, thus, they focused on interpreting accuracy results because “participants' efforts are generally focused more on performing the task accurately than responding as fast as they can” (p. 4).

In light of these findings, how is interpretation of results affected when associations for some *n*-back task performance indices can be found but not for others? For example, in two studies that either used emotional words (Kopf et al., 2013) or pictures of emotional scenes (Marx et al., 2011) it was found that accuracy (but not reaction times) differed as a function of emotional valence of the stimuli. In contrast, effects of emotional stimuli (here: faces) were only found for reaction times and not for accuracy in a recent study by Cromheeke and Mueller (2016). Yet, the authors concluded that “allocating attention to affective information improved working memory” (p. 295). It might be argued that it is at least debatable if emotional stimuli indeed affected working memory performance as participants' ability to discriminate between targets and non-targets was not influenced. What these examples illustrate is that reaction times and accuracy in *n*-back tasks should not be interpreted interchangeably. Specifically, I argue that it is not reasonable when different studies reach similar conclusions (e.g., that effects of certain stimuli on or certain group differences in working memory performance were found), although these conclusions are based on different dependent variables (e.g., on reaction times in one study and on accuracy in another study).

In addition to these considerations, what constitutes accuracy is surprisingly rarely defined in most reports and/or it includes different types of errors (e.g., Jonides et al., 1997; Carlson et al., 1998; Perlstein et al., 2003; Harvey et al., 2005; Miller et al., 2009; Dodds et al., 2011). In *n*-back tasks, participants can either correctly press a button in response to targets (= hits), incorrectly press a button in response to non-targets (= commission errors or false alarms), and incorrectly do not press a button in response to targets (= omission errors or misses). However, researchers often do not make this distinction (for an exception see, e.g., Schmidt et al., 2009). In contrast to motor inhibition tasks (e.g., Go/No-go tasks), in which the main measure of interest is commission errors (e.g., Newman et al., 1985), omission errors are more frequent than commission errors in *n*-back tasks. Of note, it appears that these two types of errors have different correlates and, thus, may represent different processes. For example, in two studies that used 2-back tasks with food and neutral pictures (Meule et al., 2012; Meule, 2016), reaction times were positively correlated with the number of omission errors [*r*_(*n* = 70)_ = 0.440, *p* < 0.001 and *r*_(*n* = 56)_ = 0.449, *p* = 0.001], but not with the number of commission errors [*r*_(*n* = 70)_ = 0.095, *p* = 0.435 and *r*_(*n* = 56)_ = 0.178, *p* = 0.188]. Omission and commission errors were unrelated [*r*_(*n* = 70)_ = 0.093, *p* = 0.443 and *r*_(*n* = 56)_ = 0.145, *p* = 0.285]. Moreover, in a study by Oberauer (2005), in which neutral words were used, only omission but not commission errors or reaction times were associated with measures of working memory capacity.

In conclusion, it is argued that researchers need to carefully interpret their findings derived from *n*-back tasks, particularly when these findings diverge depending on whether using reaction times or accuracy. Moreover, it appears necessary that researchers not only report accuracy but also differentiate between omission and commission errors. In addition, it may be preferable to report other task performance indices that are calculated from hits and false alarms such as discrimination index *d*′ and response bias *C*, as has been suggested by researchers who used modified versions of the *n*-back task (Kane et al., 2007; Haatveit et al., 2010). As the *n*-back task has been criticized for lacking clear associations with other working memory tasks (Kane et al., 2007; Jaeggi et al., 2010), using more fine-grained analyses of *n*-back task performance beyond reporting reaction times and accuracy may, therefore, reveal clearer insights about its validity as a measure of working memory performance, its neural or psychopathological correlates, and its utility in applied neuropsychology.

## Author contributions

The author confirms being the sole contributor of this work and approved it for publication.

### Conflict of interest statement

The author declares that the research was conducted in the absence of any commercial or financial relationships that could be construed as a potential conflict of interest.
